# The Southern European Atlantic Diet and depression incidence: a multicohort study

**DOI:** 10.1093/eurpub/ckac129.552

**Published:** 2022-10-25

**Authors:** A Carballo-Casla, D Stefler, R Ortolá, Y Chen, R Kubinova, A Pajak, S Malyutina, F Rodríguez-Artalejo, EJ Brunner, M Bobak

**Affiliations:** 1 Department of Preventive Medicine and Public Health, Universidad Autónoma de Madrid, Madrid, Spain; 2 CIBER of Epidemiology and Public Health, CIBERESP, Madrid, Spain; 3 Department of Epidemiology and Public Health, University College London, London, UK; 4 National Institute of Public Health, Prague, Czechia; 5 Department of Epidemiology and Population Studies, Jagiellonian University Collegium Medicum, Krakow, Poland; 6 IMDEA Food Institute, CEI UAM+CSIC, Madrid, Spain; 7 Novosibirsk, Russia

## Abstract

**Background:**

The Southern European Atlantic Diet (SEAD) is the traditional diet of Northern Portugal and North-Western Spain, but it may resemble that of other European countries. Higher adherence to SEAD has been associated with lower risk for myocardial infarction and all-cause mortality, but its relationship with mental health is uncertain. We examined the association between SEAD and depression incidence in Southern, Central, and Eastern Europe.

**Methods:**

We used data from participants ≥45 years from the Seniors-ENRICA-2 and HAPIEE cohorts, who were followed for a median time of 3.6 years. SEAD comprised fresh fish, cod, red meat and pork products, dairy, legumes and vegetables, vegetable soup, potatoes, whole-grain bread, and wine. Depressive symptoms were assessed with the GDS 10 and the CES-D 10 (participants with scores ≥4 were considered depression cases). Statistical analyses were performed among the 14675 participants who were depression-free at baseline.

**Results:**

Higher adherence to SEAD was preliminarily associated with lower depression incidence in the pooled sample (fully adjusted odds ratio [95% confidence interval] per 1-SD increment in the SEAD = 0.93 [0.89,0.98]). Results were consistent in Spain (odds ratio [95% confidence interval] 0.86 [0.68,1.08]), Czechia (0.92 [0.82,1.04]), Poland (0.94 [0.88,1.01]), and Russia (0.93 [0.87,1.00]). The association of SEAD with depression in the pooled sample was similar to that found for the Alternate Healthy Eating Index (odds ratio [95% confidence interval] per 1-SD increment = 0.94 [0.89,0.99]) and the Mediterranean Dietary Score (0.94 [0.90,0.98]).

**Conclusions:**

Adherence to SEAD was preliminarily associated with lower depression incidence in Spain, Czechia, Poland, and Russia. These findings may support the development of mental health guidelines for Southern European Atlantic populations based on their traditional diet, and for Central and Eastern European countries based on the food components of SEAD.

**Key messages:**

• Adherence to the Southern European Atlantic Diet (traditional diet of Northern Portugal and North-Western Spain) was associated with lower depression incidence in Spain, Czechia, Poland, and Russia.

• Mental health guidelines for Southern European Atlantic populations may reference their traditional diet, while those for Central and Eastern Europe could benefit from including its food components.

